# PHBV/PAM Scaffolds with Local Oriented Structure through UV Polymerization for Tissue Engineering

**DOI:** 10.1155/2014/157987

**Published:** 2014-01-22

**Authors:** Yu Ke, Gang Wu, Yingjun Wang

**Affiliations:** ^1^Department of Biomedical Engineering, College of Life Science and Technology, Jinan University, Guangzhou 510632, China; ^2^Key Laboratory of Biomaterials of Guangdong Higher Education Institutes, Jinan University, Guangzhou 510632, China; ^3^Biomaterial Research Institute, School of Materials Science and Engineering, South China University of Technology, Guangzhou 510641, China; ^4^National Engineering Research Center for Tissue Restoration and Reconstruction, Guangzhou 510006, China

## Abstract

Locally oriented tissue engineering scaffolds can provoke cellular orientation and direct cell spread and migration, offering an exciting potential way for the regeneration of the complex tissue. Poly(3-hydroxybutyrate-*co*-3-hydroxyvalerate) (PHBV) scaffolds with locally oriented hydrophilic polyacrylamide (PAM) inside the macropores of the scaffolds were achieved through UV graft polymerization. The interpenetrating PAM chains enabled good interconnectivity of PHBV/PAM scaffolds that presented a lower porosity and minor diameter of pores than PHBV scaffolds. The pores with diameter below 100 **μ**m increased to 82.15% of PHBV/PAM scaffolds compared with 31.5% of PHBV scaffolds. PHBV/PAM scaffold showed a much higher compressive elastic modulus than PHBV scaffold due to PAM stuffing. At 5 days of culturing, sheep chondrocytes spread along the similar direction in the macropores of PHBV/PAM scaffolds. The locally oriented PAM chains might guide the attachment and spreading of chondrocytes and direct the formation of microfilaments *via* contact guidance.

## 1. Introduction

Tissue engineering, being used to restore, maintain, or enhance tissues and organs, offers a potential remedy for tissue regeneration [1, 2]. Polyhydroxyalkanoates (PHAs), a class of natural polyester made by microorganisms *via* fermentation, have shown great promise for tissue engineering applications due to its biodegradability, biocompatibility, nontoxicity, and piezoelectricity [3–5]. PHAs scaffolds has been proved to support osteoblasts, osteoblast-like cells (MC3T3-E1 S14), bone marrow stromal cells or adipose stem cells to construct bone implants [6–9]. Chondrocytes or adipose stem cells being seeded on PHAs scaffolds have produced neocartilage [10, 11], while myoblastic cell lines C2C12 and H9c2 have formed skeletal myotube [12]. Khorasani et al. have studied PHAs scaffold cultured with P19 mouse embryonal cell line, showing its capacity for neural tissue engineering [13]. Among PHAs, two main commercially available polyesters are poly(3-hydroxybutyrate) (PHB) and poly(3-hydroxybutyrate-*co*-3-hydroxyvalerate) (PHBV).

Surface-dependent cell and tissue response to implants are controlled by many biological principles. The physicochemical properties of a biomaterial and its surface-texture greatly influence the cell-material interaction. In a process of contact guidance, topographical features on the surface regulate the spatial distribution of adhesive contacts and thereby determine the spreading of a cell. Then, cells reorganize extracellular matrix (ECM) to provide contact guidance that facilitates 3D migration [14]. Contact guidance of cells is highly correlated with the direction of cell migration, and provides an efficient end-point to determine whether the cells integrate the physical and chemical cues of the material [15].

Oriented scaffolds could provoke cellular orientation *via *contact guidance. Several fabrication techniques have been used to produce scaffolds with longitudinally oriented pores. For example, Uto et al. have crosslinked end-functionalized poly(*ε*-caprolactone-*co*-d,l-lactic acid) in the presence of longitudinally oriented silica fibers that function as the templates and later can be dissolved by hydrofluoric acid to produce uniaxial pores [16]. Isobe et al. have prepared various types of three-dimensional oriented collagen scaffolds by arraying highly oriented collagen string gels being drawn under a shear and extensional flow [17].

Electrospinning method has been applied for the fabrication of scaffolds, which uses an electrical charge to draw very fine fibers between two target collectors. Cells could stretch along the direction of aligned nanofibrous collagen, poly(*ε*-caprolactone), or elastin A/collagen scaffolds [18–20]. Through a layer-by-layer approach, Builles et al. have fabricated a fibrous scaffold consisting of orthogonal layers of aligned collagen fibers to mimic the organization of the corneal stroma. Epithelial cells have been found to penetrate into the scaffolds and arrange in layers, with an abrupt change in cell orientation between layers [21]. Much focus has been paid on electrospinning to produce fibrous scaffolds, but low productivity is still one of its main disadvantages.

Micropatterning has recently been proved a powerful technique for creating highly organized structures in biomaterials. Micro- and/or nanoscale ridge/groove features have been fabricated on poly(dimethylsiloxane) (PDMS) stamps by using soft lithography, which consist of a “hard” PDMS layer to retain the topographical features and a pliable PDMS layer for easy removal and handling of the stamp [22]. Chen et al. have produced a Teflon microfluidic chip using a PDMS master and prepared silica nanotubes with a highly organized structure on the chip [23]. Nanoimprint lithography has also been applied to fabricate cyclic olefin copolymer films with a grating pattern of nanoscale grooves and ridges [24]. It should be noted that micropatterning is a rather complex process. The most popular topography is the microgroove because they are easily directed by soft-/photo-lithographic techniques.

Controlled freezing and lyophilization treatment is a useful method to prepare scaffolds containing longitudinally oriented channels. Water-soluble collagen, agarose, gelatin, collagen/chitosan, hydroxyapatite or bioactive glass scaffolds, [25–30] and water-insoluble poly(d,l-lactic acid) scaffolds [31] with axially oriented pores have been fabricated by controlling ice crystals growth longitudinally in the freeze-drying process. Jia et al. have prepared oriented cartilage ECM scaffolds by using a modified temperature gradient-guided thermal-induced phase separation technique followed by the freeze-drying, which composed of microtubules being arranged in parallel in vertical section [32].

It is still very difficult to fabricate a complex oriented scaffold using both hydrophilic and hydrophobic polymers, though many developments have been achieved. In a previous study, we have fabricated PHBV/polyacrylamide (PAM) scaffolds through photografting polymerization. In the complex scaffolds, the framing PHBV scaffolds supported the scaffold's structure and resisted deformation, while gel-like PAM inside the scaffolds mimicking the structure of ECM provided a hydrophilic surrounding for cells [33]. In this work, we introduced local oriented PAM into the PHBV scaffold and tried to investigate contact guidance of chondrocytes along the oriented structure primarily.

## 2. Experimental

### 2.1. Materials

PHBV with 8% hydroxyvalerate content (number-average molecular weight = 1.85 × 10^5^, polydispersity index = 2.2) were purchased from Aldrich, USA. Acetone, chloroform and glutaraldehyde were purchased from Tianjin Chemical Reagent No. 1 Plant (China). Acrylamide and benzophenone (BP) were obtained from Shanghai Runjie Chemical (China). Sodium chloride as porogen was acquired from Tianjin Fuchen Chemical (China). Ethylene diamine-*N,N*-tetraacetic acid (EDTA) was from Weijia Biotechnology (China). Penicillin, streptomycin, trypsinase, and collagenase II were purchased from Sigma (USA). Dulbecco's Modified Eagle Medium (DMEM) was obtained from GIBCO (USA). Fetal bovine serum (FBS) was purchased from PAA (Austria).

### 2.2. Preparation of PHBV Scaffolds and PHBV/PAM Scaffolds

PHBV scaffolds were prepared through a particulate-leaching technique. 3 g of PHBV was dissolved in chloroform, followed by addition of sieved NaCl (27 g, 200 ~ 300 *μ*m). The suspension was sonicated for 20 minutes and pressed tightly into a column glass mold. The samples were then dried at 25°C for 24 hours to release chloroform. The resulting solid PHBV column was dialyzed against distilled water for 72 hours to remove NaCl and vacuum-dried for 48 hours. After freezing treatment in liquid nitrogen for about 30 seconds, the PHBV column was immediately cut into PHBV scaffolds with a diameter of 10 cm and a height of 10 cm.

Before preparing PHBV/PAM scaffolds, the PHBV scaffolds were soaked into 5 wt% BP solution with acetone as solvent for 6 hours and then dried at 25°C to remove acetone. The BP-preabsorbing scaffolds were put into 10 wt% aqueous acrylamide under three cycles of evacuation/repressurization purging. When no bubble emerged from the aqueous acrylamide, the PHBV scaffolds being sunk in the bottom were collected and dipped in acrylamide solution being contained in a small reactor. The reactor was placed under a high-pressure mercury lamp (Philips 400S) for about 65 minutes. After UV photopolymerization, the scaffolds were Soxhlet extracted with acetone for 96 hours and rinsed with deionized water till constant weight. The final PHBV/PAM scaffolds were freeze-dried for 48 hours before use.

### 2.3. Characterization

#### 2.3.1. FTIR Spectra

Fourier transformed infrared spectroscopy (FTIR) spectra were recorded on a Bruker (Germany) Vector 33 infrared analysis equipped with an attenuated total reflectance (ATR) accessory providing analysis of the surface. The spectra comprised 16 scans measured at a resolution of 4 cm^−1^ in the 4000~600 cm^−1^ range.

#### 2.3.2. Morphology

Morphologies were observed with a Philips (Holland) XL 30 scanning electron microscopy (SEM). Scaffolds were snapped after being plunged into liquid nitrogen for about 1~2 minutes. The cut samples were mounted on metal stubs and coated with gold through a Hitachi (Japan) E-1010 sputter. An energy dispersive X-ray spectrometer (EDX) being attached was used to analyze the component.

#### 2.3.3. Pore Size

Pore size distribution of the scaffolds was studied through a Quantachrome (USA) PoreMaster 33 mercury intrusion porosimetry (MIP). Pore diameter (*D*) was calculated according to the applied pressure (*P*) by using the Wasburn equation:

(1) $$\begin{array}{c} D=-\frac{4\gamma \cos\theta }{P}, \end{array}$$

where *γ* and *θ* are the hydrargyric surface tension and contact angle on solid surface, respectively.

#### 2.3.4. Porosity

Porosity of the scaffolds was measured by the mass method using ethanol as the displacement liquid. A dry scaffold (*W*_*s*_) was immersed in ethanol under evacuation-repressurization cycles until no air bubbles emerged. The ethanol-impregnated scaffold and the container with ethanol were weighed as *W*_*a*_, while the container with ethanol was weighed as *W*_*b*_ after the scaffold was removed into a cylinder. Ethanol was filled in the cylinder to a predetermined graduation. The ethanol-impregnated scaffold and the cylinder with ethanol were weighed as *W*_2_. Then, the scaffold was removed and ethanol was refilled to the predetermined graduation. The weight of the cylinder with ethanol was recorded as *W*_1_. Porosity (*ε*) and density (*ρ*_*s*_) were defined by the following equations, respectively, the mean value was calculated on five different samples:

(2) $$\begin{array}{c} \varepsilon =\frac{W_{a}-W_{b}-W_{s}}{\left(W_{a}-W_{b}\right)-\left(W_{2}-W_{1}\right)},\\ \rho_{s}=\frac{\rho W_{s}}{\left(W_{a}-W_{b}\right)-\left(W_{2}-W_{1}\right)}, \end{array}$$

where *ρ* was the density of ethanol.

#### 2.3.5. Compressive Properties

Compressive properties of the scaffolds were performed on an Instron 5567 mechanical tester (USA) with a speed of 1 mm/min. Column-shaped scaffolds measuring 10 mm in diameter and 10 mm in height were used. The compressive load and extension curve was graphed. Wet samples were immersed in deionized water for 24 hours prior to the measurement. The data were the average of seven scaffolds.

#### 2.3.6. Cell Studies

Chondrocytes were harvested from knee joints of male sheep weighing 17 kg (six-month-old, Guanhao Biotech, China). NIH Guide on animal experimentation was strictly followed. Cartilage slices were incised from the patellar groove and placed in phosphate buffered saline containing penicillin (100 mg/L) and streptomycin (100 mg/L). The slices were then exposed to 0.25% trypsinase at 37°C for 30 minutes, rinsed and digested with 0.2% collagenase II in DMEM without FBS at 37°C and 5% CO_2_. Four hours later, the cells were collected every 2 h and the cell suspension was filtrated and centrifuged at 1000 rpm for 5 minutes. The cell pellet was resuspended in DMEM containing 20% FBS. 5 mL of Cell suspension (2 × 10^5^ cells/mL) was seeded in a 25 cm^2^ polystyrene plate. Culture medium was initially replaced 48 hours later and then changed every 2 days. At 80~90% confluence, cells were passaged with 0.25% trypsinase supplemented with 0.02% EDTA.

Second-passage chondrocytes were trypsinized at a density of 2 × 10^5^ cells/well were seeded evenly on PHBV and PHBV/PAM scaffolds that had been sterilized by exposure to epoxyethane vapor. The cell-seeded scaffolds were incubated at 37°C under 5% CO_2_. At 5 days of culture, the cells being attached were fixed with 2.5% glutaraldehyde for 30 minutes. The cell-scaffold complex was washed, dehydrated by slow water replacement using series of ethanol solution (30, 50, 70 and 90%) for 10~15 minutes, and dried at critical temperature. The samples were then mounted on metal stubs and coated with gold for SEM analysis.

## 3. Results 

### 3.1. Chemical Composition

**Figure 1:**
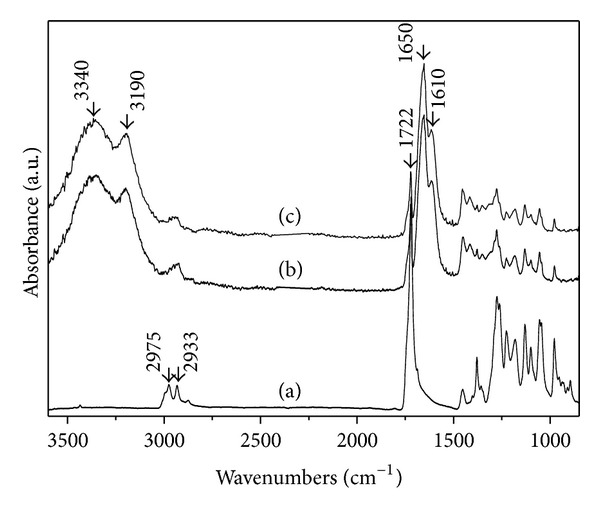
ATR-FTIR spectra of the scaffolds: (a) Surface, PHBV scaffold; (b) Surface with irradiation, PHBV/PAM scaffold; (c) Surface without irradiation, PHBV/PAM scaffold.

ATR-FTIR spectra have been used to illustrate chemical composition of the surface of the scaffolds. As shown in Figure 1, the absorbance peaks at 2975, 2933, and 1722 cm^−1^ were asymmetric and symmetric stretching vibration of CH_3_ and stretching vibration of C=O of PHBV, respectively. The peaks at 3340 and 3190 cm^−1^ belonged to asymmetric and symmetric stretching vibration of N–H, respectively. The peaks at 1662 and 1610 cm^−1^ were attributed to the strong stretching vibration of C=O (Amide I) and the medium bending vibration of N–H (Amide II), respectively. The latter four peaks were characteristic absorbance peaks of acrylamide groups, attributed to the PAM chains being grafted on the surface of PHBV/PAM scaffold (Figure 1(b)) [33]. It could be seen that these four absorbance peaks were also shown on the surface without irradiation (Figure 1(c)).

### 3.2. Morphology

**Figure 2:**
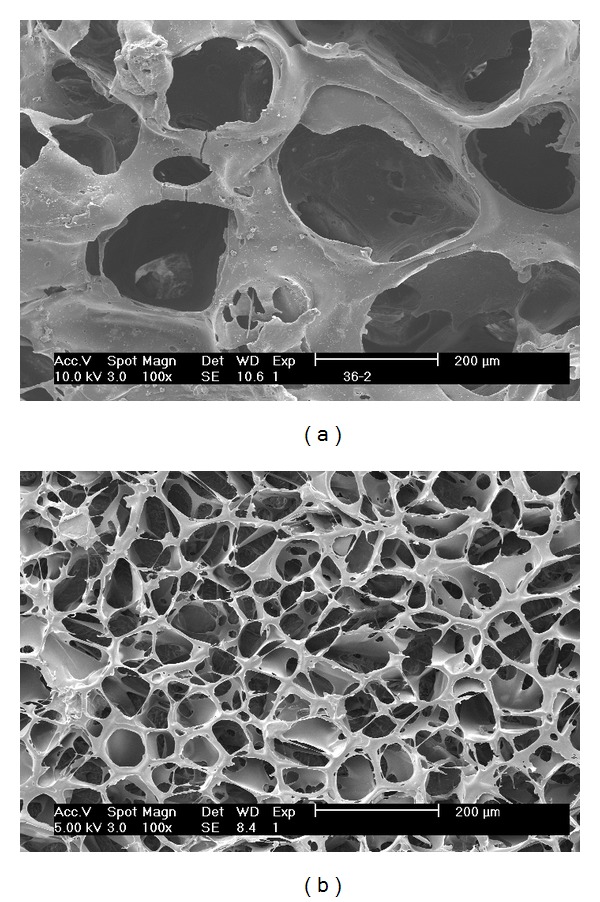
Surface SEM images of scaffolds: (a) PHBV scaffold; (b) PHBV/PAM scaffold.

Surface morphology of the scaffolds was shown in Figure 2. The high interconnective PHBV scaffolds possessed macropores with a diameter up to 300 *μ*m. Many micropores with different size and shape were located on the walls of the macropores. PHBV/PAM scaffolds showed a higher interconnectivity than PHBV scaffolds. A web layer with cavities of various sizes was formed *via* freeze-drying treatment of the grafted PAM chain, as already confirmed in ATR-FTIR study.

**Figure 3:**
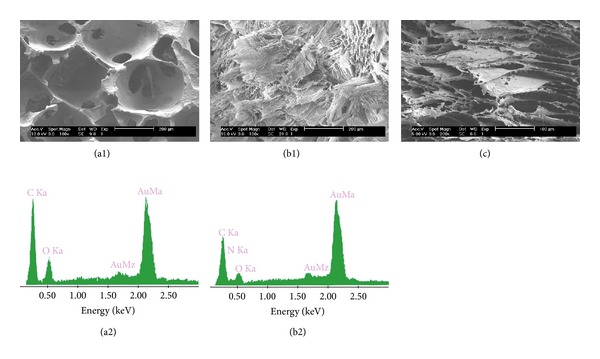
Cross-section SEM images and EDX spectra of scaffolds: (a1, a2) PHBV scaffold, in cross-sectional orientation; (b1, b2) PHBV/PAM scaffold, in cross-sectional orientation; (c) PHBV/PAM scaffold, in longitudinal orientation.

Figure 3 illustrates cross-sectional morphology of the scaffolds. PHBV scaffolds (Figure 3(a1)) showed porous microstructure with a high degree of interconnectivity. The pores were nearly round-shaped with an average diameter of ~200 *μ*m. The walls of different thickness were dotted with small holes. The border of the walls formed irregular cavities that might be larger than the macropores or smaller than the micropores. Some cavities were filled with PHBV substrate to form a ridge structure. The high interconnectivity *via* micropores was suitable for the exchange of nutrient and metabolic waste. EDX results of the cross-section showed that PHBV scaffolds mainly consisted of carbon and oxygen atoms (Figure 3(a2)).

PHBV/PAM scaffolds possessed lower porosity and much smaller pore size than PHBV scaffold. The macropores were stuffed by locally orienting slices (Figure 3(c)); however, the border of the walls remained (Figure 3(b1)). The cavities along the border of the walls disappeared, perhaps being filled with the PAM chains. EDX results showed that the slices were composed of carbon, oxygen, and nitrogen atoms (Figure 3(b2)). Nitrogen was attributed to the PAM chains that had been introduced in the macropores of PHBV scaffolds (Figure 3(c)).

### 3.3. Pore Size and Porosity

**Figure 4:**
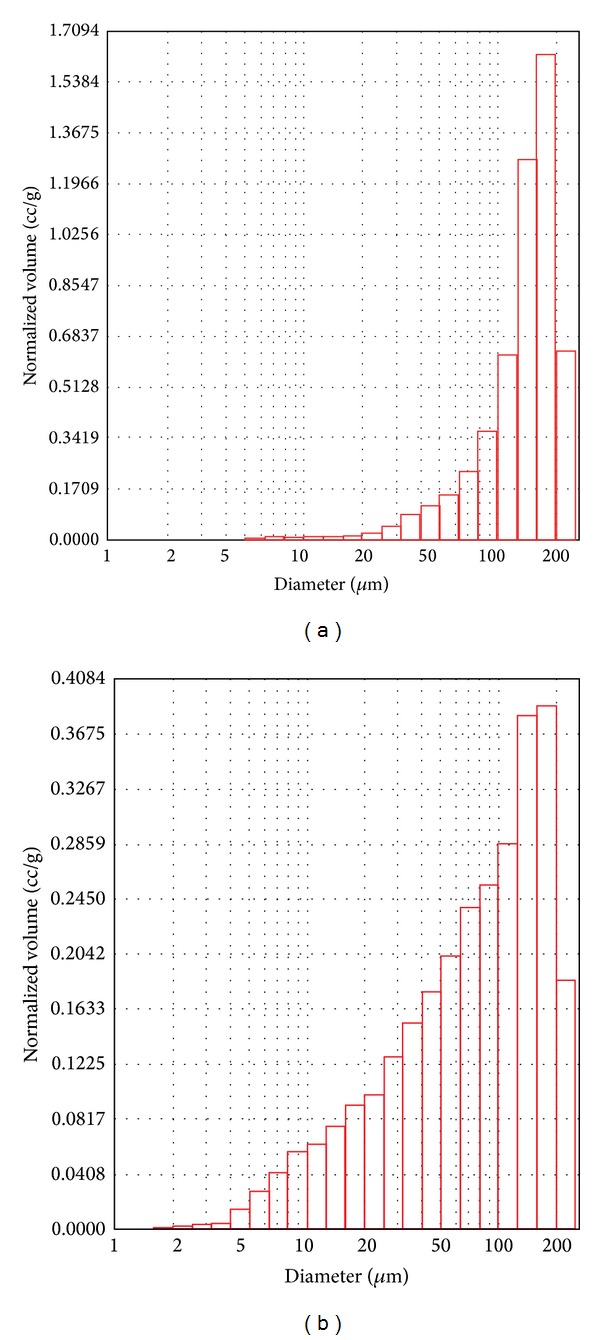
Representative plots of normalized intrusion volume *versus* pore diameter of scaffolds: (a) PHBV scaffold; (b) PHBV/PAM scaffold.

Figure 4 presents pore size distribution curves of the scaffolds by using MIP measurement. Owing to the limitation of the lowest filling pressure for MIP measurement, the pore sizes over 240 *μ*m cannot be read. The maximum normalized volume of PHBV/PAM scaffold was much smaller than that of PHBV scaffold. The pores with diameter below 50 *μ*m were constituted 9.80% and 37.42%, respectively, for PHBV and PHBV/PAM scaffolds. The pores with diameter above 100 *μ*m decreased from 68.50% of PHBV scaffold to 17.85% of PHBV/PAM scaffold accordingly. The mean diameter of PHBV/PAM scaffold was 75.15 *μ*m, about 61% of PHBV scaffold. The porosity of PHBV/PAM scaffold was 52.4%, nearly 57% of PHBV scaffold, while the density of PHBV/PAM scaffold was 0.41 g/cm^3^, 3.4 times of the PHBV scaffold (Table 1). The results confirmed that the pore size and porosity of PHBV/PAM scaffolds decreased by the stuffing effect of the PAM chains.

### 3.4. Compressive Properties

A compression curve of an ideal porous material consists of three stages: the initial elastic stage reflects the bending of the walls of the pores under the compressive load; in the intermediate stage, the stress increases slowly and the pores begin to collapse because of the plastic buckling, plastic hinge, or even brittle fracture of the walls; in the final stage, the stress increases sharply and the pores collapse completely, illustrating the densification of porous material. The compressive curve of the scaffolds is more complicate than that of the porous material due to uncertain distortion under the compressive load. Usually, the scaffolds are characterized by an unclear initial elastic stage and decreasing stain at the beginning of the densification.

**Figure 5:**
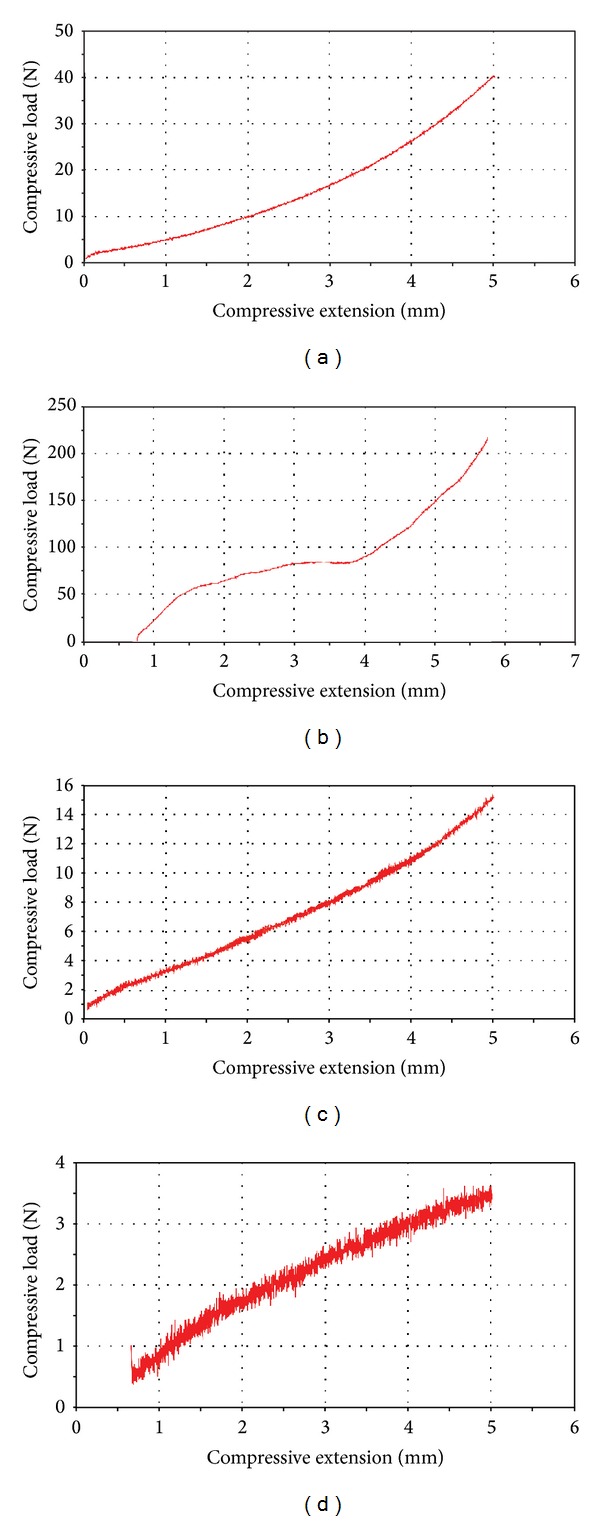
Compressive load and extension curves of the scaffolds: (a) dry PHBV scaffold; (b) dry PHBV/PAM scaffold; (c) wet PHBV scaffold; (d) wet PHBV/PAM scaffold.

The compressive load and extension curves of the scaffolds were shown in Figure 5. The Dry PHBV scaffold showed a modulus of 0.26 Mpa in the initial stage, followed by the densification stage (Figure 5(a)). However, the dry PHBV/PAM scaffold illustrated three-stage deformation under the compressive load, with an elastic modulus of 4.59 Mpa (Figure 5(b)). There were no distinct stages in the compressive load and extension curves of the wet scaffolds. Under the compressive load, water contained in the wet PHBV scaffold was excluded. Therefore, the stress of the wet PHBV scaffold decreased compared with that of the dry PHBV scaffold. The elastic modulus for the wet PHBV scaffold was 0.19 Mpa (Figure 5(c)). It should be noted that the walls of the pores might have collapsed or fractured similarly to those of dry PHBV scaffolds at the same stain. In the curve of the wet PHBV/PAM scaffold, no loading was recorded in the beginning, owing to the water exclusion from the swollen PAM chains being grafted on the surface of PHBV/PAM scaffold. The wet PHBV/PAM scaffold presented an elastic deformation under the compressive load, with the modulus of 0.04 Mpa (Figure 5(d)).

The average compressive properties of the scaffolds were summarized in Table 2. The stress of PHBV/PAM scaffolds at 5 mm of compressive extension was nearly four times than that of PHBV scaffold at dry condition, however, only 20% at wet condition. The dry PHBV/PAM scaffold showed a much higher elastic modulus than the dry PHBV scaffold, but the wet PHBV/PAM scaffold possessed a lower elastic modulus than the wet PHBV scaffold.

### 3.5. Cell Studies

**Figure 6:**
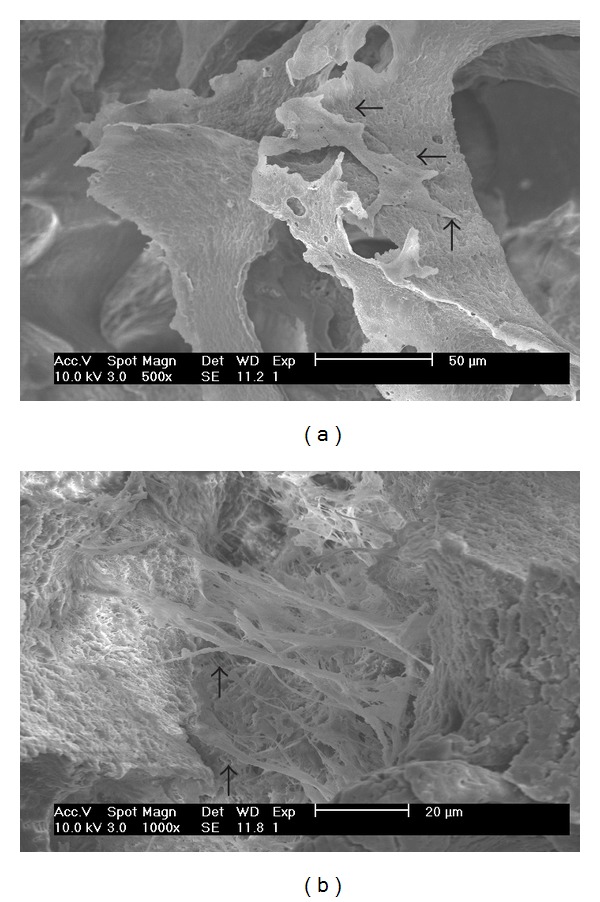
SEM photograph of chondrocyte-scaffolds at 5 days of culturing: (a) PHBV scaffold; (b) PHBV/PAM scaffold.

Chondrocytes morphology in the scaffolds at 5 days of culturing was shown in Figure 6. In PHBV scaffolds, chondrocytes adhered to the wall of the inner macropores and protruded short pseudopodia to the surface of the wall, as marked by arrows. In PHBV/PAM scaffolds, chondrocytes extended long and tiny pseudopodia to the opposite wall of inner macropores. Most of the chondrocytes stretched along the similar direction in the macropores of scaffolds. We presumed that the oriented PAM gels imposed restriction on the formation of thin bundles of microfilaments *via* the contact guidance process.

## 4. Discussion

PHBV/PAM scaffolds were obtained through photografting polymerization, where BP as photo-initiator was activated under UV light, and abstracted the tertiary hydrogen from PHBV on the surface of the scaffold by inelastic collision. Ketyl radical (BPH*) dimerized, while polymer radical (P*) formed and reacted with the vinyl groups of acrylamide in solution to initiate graft polymerization effectively on the surface [34, 35]. In order to increase the amount of polymer radicals formed in heterogeneous reaction system, BP-preabsorbing treatment was applied to the scaffolds before UV irradiation.

The PAM gels inside the scaffolds might be formed owing to chains transfer reaction and/or PAM homo-polymerization. The reactive PAM chains may abstract hydrogen of PHBV chains or nearby PAM grafted chains, so that the newly formed PHBV or PAM radicals might react with acrylamide to initiate grafting polymerization. Therefore, PAM had been introduced into PHBV scaffolds [33]. Different from our previous study, we employed the evacuation-repressurization cycles to BP-treating scaffolds. Aqueous acrylamide was introduced inside scaffolds, so as to facilitate the *in situ* polymerization with PHBV or PAM radicals. In addition, BP being absorbed inside the scaffolds would be suspended in the aqueous acrylamide and initiated to form homo-PAM, interlacing with grafted PAM chains. The PAM chains propagated, penetrated along the pores, and finally reached the surface without irradiation. Because large amounts of aqueous acrylamide were introduced inside the macropores *via* the evacuation-repressurization cycles, a well local oriented gel structure was formed inside the macropores.

Precise control of pore geometry, size, orientation, and interconnectivity of the scaffolds is essential to the fabrication technique. The interconnected macropores are considered as a requirement for scaffolds based on the size and migration of cells, while micropores and mesopores are most useful for solute diffusion [36, 37]. The porosity and mean diameter of PHBV/PAM scaffolds decreased while compared with those of PHBV scaffolds, and fraction of pores below 100 *μ*m increased. But the grafted hydrophilic PAM chains were beneficial to the nutrient diffusion. The oriented structure was also favorable for infiltrating cells into the scaffolds and allowed adequate contact between the scaffolds and surrounding cells *via *contact guidance.

As application in tissue engineering is concerned, the internal macropores are required to be open with good interconnectivity. Because NaCl content for fabricating PHBV scaffold was about 90 wt%, the resulting pores would be interconnective due to geometrical packing [38]. Interconnectivity of PHBV scaffold is important for the preparation of PHBV/PAM scaffolds. Poor interconnectivity would retard the propagation of polymerization, so that the PAM chains would accumulate locally inside the scaffolds and finally led to the collapse of PHBV/PAM scaffold. PHBV scaffolds with good interconnectivity would produce an interpenetrating PAM chains through the PHBV scaffolds. Once the UV irradiation time increased, the PAM chains would accumulate on the surface without irradiation. The resulted PHBV/PAM scaffolds retained their shape, but the height increased due to the grafted PAM chains on both surfaces. The swollen PAM chains formed a continuous network inside PHBV scaffold, which would leave a connective pore structure after freeze-drying treatment.

Scaffolds structures, including morphology, pore size, and porosity, determined the final properties. Because of their higher density and lower porosity, the PHBV/PAM scaffolds possessed higher modulus than PHBV scaffolds at dry condition. While immersed in water, the PHBV functioned as the frame of the scaffolds, and the inner hydrogels provided routes for nutrients transportation and guided cells orientation. Our previous studies showed that chondrocytes adhered to the surface PAM-modified PHBV scaffolds or the walls of the pores, spreading in spindle-shape with long extension [39]. However, the lamellar PAM networks facilitated the cells orientation and spreading along the local oriented PAM gels inside the macropores* via* contact guidance.

## 5. Conclusions

PHBV/PAM scaffolds with locally oriented gel structure have been achieved *via* UV graft polymerization. The hydrophobic PHBV and hydrophilic PAM provided the biomacromolecules or cells with different surface that might induce different physicochemical reaction. The locally oriented PAM favored the development of cells and guided the attachment and spreading of cells along similar direction, signifying an orderly tissue inside the macropores of scaffolds *in vitro*. These oriented PHBV/PAM scaffolds might form an orientated structure in short tubes, which showed potential applications for the regeneration of complex tissue.

## Figures and Tables

**Table 1:** Porosity and density of PHBV and PHBV/PAM scaffolds.

Sample	Porosity (%)	Density (g/cm^3^)
PHBV	91.8 ± 2.3	0.12 ± 0.04
PHBV/PAM	52.4 ± 4.6	0.41 ± 0.58

**Table 2:** Compressive properties of PHBV and PHBV/PAM scaffolds.

Sample	Stress^a^ (Mpa)	Modulus (Mpa)
Dry PHBV	0.49 ± 0.04	0.23 ± 0.09
Dry PHBV/PAM	2.52 ± 0.41	4.64 ± 0.31
Wet PHBV	0.18 ± 0.02	0.15 ± 0.04
Wet PHBV/PAM	0.04 ± 0.01	0.05 ± 0.03

^a^Stress at 5 mm of compressive extension.
